# Impurity bound states in fully gapped *d*-wave superconductors with subdominant order parameters

**DOI:** 10.1038/srep44107

**Published:** 2017-03-10

**Authors:** Mahdi Mashkoori, Kristofer Björnson, Annica M. Black-Schaffer

**Affiliations:** 1 Department of Physics and Astronomy, Uppsala University, Box 516, SE-751 20 Uppsala, Sweden

## Abstract

Impurities in superconductors and their induced bound states are important both for engineering novel states such as Majorana zero-energy modes and for probing bulk properties of the superconducting state. The high-temperature cuprates offer a clear advantage in a much larger superconducting order parameter, but the nodal energy spectrum of a pure *d*-wave superconductor only allows virtual bound states. Fully gapped *d*-wave superconducting states have, however, been proposed in several cuprate systems thanks to subdominant order parameters producing *d* + *is*- or *d* + *id*′-wave superconducting states. Here we study both magnetic and potential impurities in these fully gapped *d*-wave superconductors. Using analytical T-matrix and complementary numerical tight-binding lattice calculations, we show that magnetic and potential impurities behave fundamentally different in *d* + *is*- and *d* + *id*′-wave superconductors. In a *d* + *is*-wave superconductor, there are no bound states for potential impurities, while a magnetic impurity produces one pair of bound states, with a zero-energy level crossing at a finite scattering strength. On the other hand, a *d* + *id*′-wave symmetry always gives rise to two pairs of bound states and only produce a reachable zero-energy level crossing if the normal state has a strong particle-hole asymmetry.

It is well-established that a single magnetic impurity induces so-called Yu-Shiba-Rusinov (YSR) bound states inside the energy gap of conventional *s*-wave superconductors^1^,^2^,^3^. In systems with spin-orbit coupling, such intra-gap bound states have recently been proposed to give rise to emergent Majorana zero-energy modes at the end-points of chains of magnetic impurities^4^,^5^,^6^. This has lead to a surge of interest in impurity-induced bound states in superconductors, with both experimental and theoretical work focusing on properties ranging from high angular momentum scattering and complex internal structure of the impurities to quantum phase transitions and spontaneous current generation, as well as many other aspects^7^,^8^,^9^,^10^,^11^,^12^,^13^,^14^,^15^,^16^. In addition, the physical properties of an impurity give valuable information about the bulk itself and can thus be a decisive probe for establishing the properties of the bulk superconducting state^17^,^18^,^19^.

One severely limiting factor in all these studies is the low superconducting transition temperature accompanied by the small energy gap associated with conventional *s*-wave superconductors. The cuprate superconductors with their much high transition temperatures would here be a tantalizing option, if it were not for their *d*-wave order parameter symmetry which enforces a nodal energy spectrum^20^. The low-energy nodal quasiparticles prevent impurity bound states and thus a pure *d*-wave superconductor can only host virtual bound states^7^,^21^. However, in small islands of YBa_2_Cu_3_O_7−*δ*_ a fully gapped spectrum has recently been discovered and attributed to the existence of subdominant order parameters, with the superconducting symmetry likely being either 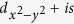-wave (d + is) or chiral 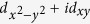-wave (*d* + *id*′)^22^,^23^. Both of these subdominant orders produce a hard gap and spontaneously break time-reversal symmetry, since the free energy very generally is minimized for subdominant parameters with an overall *π*/2-phase shift relative to the dominant order. The *d* + *id*′-wave state is also a chiral state with its non-trivial topology classified by a Chern number *N* = 2^24^. Evidence also exists that surfaces, especially the (11) surface^25^, as well as certain impurities^26^ also spontaneously generate a time-reversal symmetry breaking superconducting state with either *d* + *is*- or *d* + *id*′-wave symmetry.

In this work we establish the properties of both potential and magnetic impurities in these two fully gapped *d*-wave superconductors. More specifically, we investigate the intra-gap bound states due to potential and magnetic impurities using both an analytic continuum T-matrix formulation and numerical tight-binding lattice calculations. We show that impurities create entirely different bound states in *d* + *is*-wave and chiral *d* + *id*′-wave superconductors, despite both being fully gapped and with a dominant parent *d*-wave state. For a *d* + *is*-wave superconductor we find that a potential impurity does not induce any bound states, while a magnetic impurity gives rise to a pair of bound states, which behaves very similar to the YSR bound states in conventional *s*-wave superconductors. This includes the behaviour of the energy spectrum when tuning the scattering strength *U*_*mag*_ of the magnetic impurity. Since the *d* + *is*-wave state is topologically trivial and with a low-energy *s*-wave gap, this resemblance with a conventional *s*-wave superconductor is very plausible. More specifically, we find that the magnetic impurity bound states have a zero-energy level crossing at a finite critical scattering 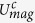. We are able to extract an analytical expression for the critical coupling which depends only on the ratio of the dominant *d*-wave to the subdominant *s*-wave order parameter. Moreover, through self-consistent tight-binding calculations we find a first-order quantum phase transition at 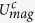, which also induces a local *π*-phase shift for the *s*-wave component of the order parameter.

For the chiral *d* + *id*′-wave state we find a very different behaviour. Here both potential and magnetic impurities induce two pairs of bound states. For superconductors with a particle-hole symmetric normal state, the bound states are two-fold degenerate and there is no level crossings for any finite coupling. Instead, it is only in the unitary scattering limit (*U*^*c*^ → ∞) that the bound states approach the middle of the gap. Doping the normal state away from particle-hole symmetry, the degeneracy is lifted for a magnetic impurity but not for a potential impurity. Moreover, for finite doping there is now a zero-energy level crossing, but for low doping it occurs only at very large scattering strengths. Self-consistent calculations for a single impurity in a *d* + *id*′-wave superconductor finds a first-order phase transition at the level crossing, but no local phase shifts in either the dominant or subdominant order parameters. Considering that recent experiments have demonstrated access to adjustable magnetic scattering strengths *U*_*mag*_^9^, magnetic impurities offer a very intriguing way to clearly distinguish between the chiral *d* + *id*′-wave state and the likewise time-reversal symmetry breaking but topologically trivial *d* + *is*-wave state.

These results have several important consequences. In spite of impurities only generating localized imperfections to the lattice structure, we find that they are very suitable for probing and differentiating the symmetry of the superconducting state. Our results show that the symmetry of the order parameter can even be probed by simply counting the number of induced subgap states. This is in sharp contrast to the virtual bound states in the pure *d*-wave state, which persist even above the superconducting transition temperature and consequently, can not be considered to be a good probe of the symmetry of the superconducting state^27^. Moreover, understanding the behaviour of single impurities is the inevitable first step for studying impurity wires or even larger impurity domains, with the aim of constructing non-trivial topological phases with Majorana fermion boundary modes. Here fully gapped *d*-wave superconductors offer a tantalizing alternative due to potentially much higher superconducting transition temperatures. Our results show that magnetic impurities in both *d* + *is*- and *d* + *id*′-wave superconductors are interesting systems in this regard, as they generate zero-energy impurity states with no band degeneracies.

## Results

### Analytic T-matrix calculations

Impurity-induced bound states only exist in *d*-wave superconductors with a fully gapped energy spectrum. Introducing a subdominant superconducting order parameter will achieve this, since it very generally align with a complex *π*/2 phase relative to the dominant *d*-wave state. Here we consider a two-dimensional (2D) 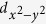-wave superconducting state with a complex subdominant order parameter such that the order parameter takes the form of 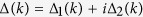 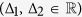. More specifically, we treat the two most likely candidates: 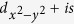
- and 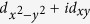-wave symmetries. In order to achieve a good analytical understanding of the effect of impurities we here first perform T-matrix calculations. Later, we confirm and extend these results by also performing self-consistent tight-binding lattice calculations.

The Hamiltonian in the presence of a single impurity (magnetic and/or potential) reads (using *ħ* = 1)


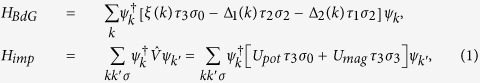


where we use the Nambu space spinor 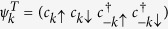. Here *U*_*pot*_ and *U*_*mag*_ are the potential and magnetic scattering matrix elements induced by the impurity, while the kinetic energy is *ξ(k*). The exact form of *ξ(k*) is unimportant as we can linearise the spectrum around the Fermi level, setting 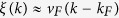. Most contributions to the superconducting state come from quasiparticles very near the Fermi level. This allows us to linearise the spectrum, sum only over states close to the Fermi level, and assume no notable dependence on |*k*| for the order parameter. The dominant order parameter is Δ_1_(*k*), while Δ_2_(*k*) represents the subdominant order parameter. We assume that the magnetic moment is large enough to ignore quantum fluctuations and thus we treat the impurity as a classical spin. The local moment of the impurity is directed along the *z* easy axis, but it is straightforward to show that the results are not affected by this assumption, since the electrons pair in the spin-singlet channel. This is different from magnetic impurities in *p*-wave superconductors, where the direction of the impurity generally affects the bound state energy^14^. The matrices *τ*_*i*_ and *σ*_*i*_ are the Pauli matrices acting in particle-hole and spin spaces, respectively, while *τ*_0_ and *σ*_0_ are unit matrices. The bare Green’s function for the superconductor is


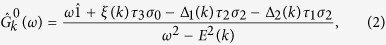


with the energy spectrum 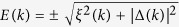. The Green’s function in the presence of a single impurity then reads 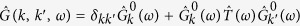, with the T-matrix 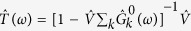
28. Therefore, finding the roots of the denominator of the T-matrix gives the energy of the impurity-induced bound states. For these analytical calculations we assume that the order parameter does not notably depend on the *magnitude* of the wave vector *k*, but only on its direction 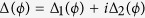, but note that this assumption is not needed in the numerical lattice calculations.

Let us first consider a fully particle-hole symmetric spectrum for the normal state, which imposes 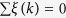. To access the T-matrix denominator the summation over the bare Green’s function is needed, 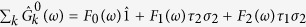, where we have defined *F*_*i*_(*ω*) as


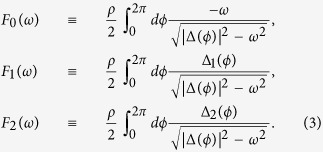


Here *ρ* = *k*_*F*_/(2*πν*_*F*_) is the density of states of the 2D free electron gas at the Fermi level. The above result is for a particle-hole symmetric normal state, but this symmetry is often broken in reality. We use the chemical potential *μ* to measure the degree of particle-hole symmetry breaking in the energy spectrum, thus leaving the case *μ* = 0 to represent full particle-hole symmetry. Considering a small deviation from particle-hole symmetry, such that 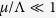 where 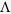 is the energy integration cut-off, the summation of the bare Green’s function also contains the term *F*_3_*τ*_3_*σ*_0_. Up to first order in 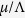 we find 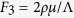, while it is straightforward to show that in this limit *F*_0_, *F*_1_, and *F*_2_ remain unchanged from the particle-hole symmetric case.

### *d* + *is*-wave state

First, we consider the 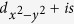 state where the order parameter is of the form 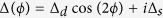. In this case *F*_1_(*ω*) = 0, due to the periodicity of the cosine function and the subdominant order parameter Δ_*s*_ not depending on *ϕ*. Then, in the limit of *μ* = 0, the bound states can be found as the solutions to


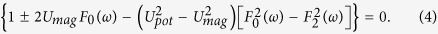


**Figure 1:**
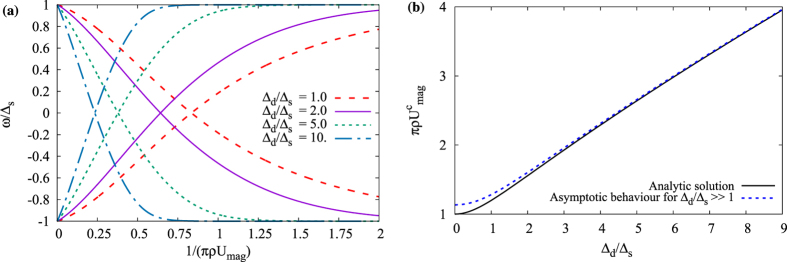
The energy spectrum of all intra-gap bound states for a magnetic impurity in a *d* + *is*-wave supercondutor as a function of 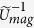 (**a**) and the critical magnetic scattering 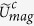 for the zero-energy level crossing as function of 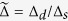 (**b**).

Since we are interested in real bound states, we only look for solutions 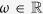. Further, to make sure that the bound states are isolated from the continuum spectrum of the superconducting quasiparticles, we limit ourselves to solutions that lie inside the gap, i.e. 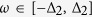. For a purely potential impurity we find no bound states, while for a purely magnetic impurity there is one pair of solutions that do not depend on the sign of *U*_*mag*_. In order to find the bound state energies for a magnetic impurity we rephrase *F*_0_(*ω*) and *F*_2_(*ω*) in terms of the complete elliptic integral of the first kind *K*^29^, resulting in


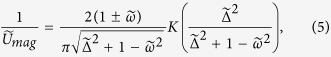


where we have defined 
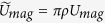
, 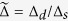, and 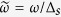. For 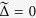, we naturally recover the YSR bound states found in a conventional *s*-wave superconductor: 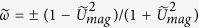
1,2,3. The bound state spectrum for a general *d* + *is*-wave superconductor comes as the solution of Eq. (5) and is illustrated in Fig. 1(a). As the figure shows, there is one pair of intra-gap bound states appearing at the gap edges for a weak magnetic impurity and moving toward the middle of the gap, such that at a critical magnetic scattering 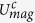 a level crossing occurs. This behaviour is qualitatively similar to the YSR bound pair in a conventional *s*-wave superconductor, where the level crossing signals a quantum phase transition between two different ground states. For a magnetic impurity stronger than 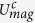, the ground state will have one unpaired electron because it is energetically favoured by the system to break a Cooper-pair to partially screen the impurity^9^,^30^. The same quantum phase transition takes place also in the *d* + *is*-wave superconductor. Interestingly, for a *d* + *is*-wave superconductor, the critical coupling depends only on 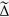 and not on Δ_*d*_ and Δ_*s*_ separately. By setting 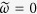 in Eq. (5), we find the analytically exact expression 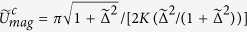. Assuming 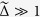, the critical coupling reads 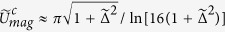, which, as seen in Fig. 1(b), only deviates at small 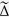 from the exact result. This clearly illustrates that, in order to find zero modes, a larger moment and/or coupling is needed when the subdominant *s*-wave parameter is small compared to the *d*-wave order.

If we now break the electron-hole symmetry of the normal state, i.e. assume 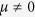, the bound states are instead found as the solution of


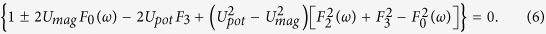


It is clear that also in this case, for a purely potential impurity there are no real roots and consequently, potential impurities never induce any bound states in a *d* + *is*-wave superconductor. Moreover, the modifications of the bound state spectrum induced by a magnetic impurity is of the order 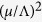 and the change to the critical coupling is also of the same order of magnitude and thus negligible. In fact, critical coupling in this approximation reads 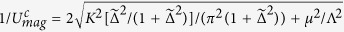.

### *d* + *id*′-wave state

Next, we turn to the impurity bound state formation inside the gap of a chiral *d*-wave or 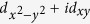-wave superconductor. Here the order parameter is 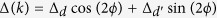. Because of the periodicity of cos(2*ϕ*) and sin(2*ϕ*), all off-diagonal terms in the summation of the bare Green’s function, i.e. *F*_*i*_ with 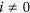, vanish. Left for a particle-hole symmetric normal state spectrum (*μ* = 0) is then only


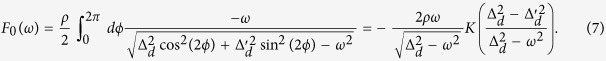


In this case the bound states are found as the solutions to


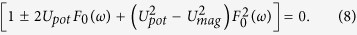


**Figure 2:**
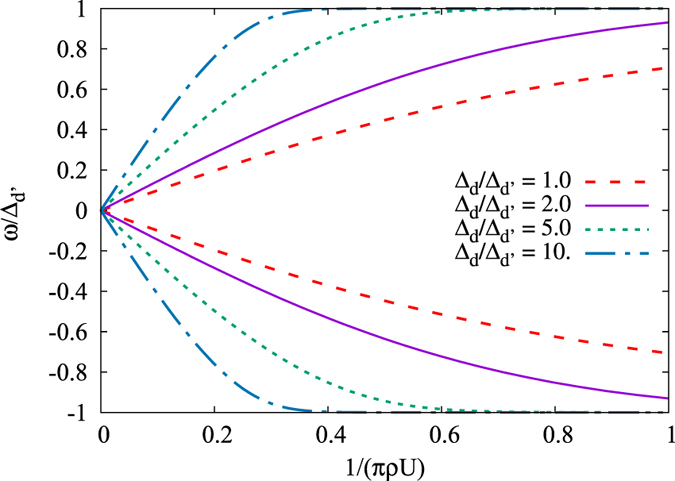
The energy spectrum of all intra-gap bound states for a purely magnetic or potential impurity in a chiral *d*-wave superconductor as a function of 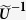, representing either a magnetic or potential scattering matrix element.

Very interestingly, pure potential or pure magnetic impurities in a chiral *d*-wave superconductor lead to exactly the same pairs of intra-gap bound states, these states are explicitly shown in Fig. 2. In earlier work it has been claimed that the number of bound states for a potential impurity is only two^8^. However, according to our results, these bound states are doubly degenerate, and there are in total actually four bound states. This statement is valid for both potential and magnetic impurities. Staying at *μ* = 0 we find that for a potential impurity the negative energy branch (those occupied at zero temperature) consist of one spin-up and one spin-down state, and there is thus a Kramers degeneracy of the states. However, for a magnetic impurity the bound states with negative energy are both spin-down quasiparticles. For an impurity with both potential and magnetic scattering effects on the charge carriers (*U*_*pot*_ and *U*_*mag*_ both non-zero), four non-degenerate bound states are generally present in a chiral *d*-wave superconductor, such that the two-fold degeneracy in Fig. 2 is lifted.

**Figure 3:**
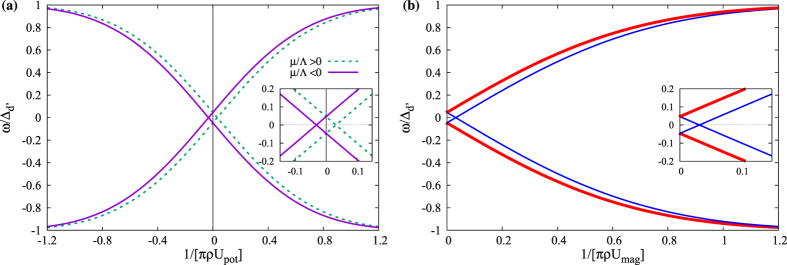
The energy spectrum of all intra-gap bound states for a potential (**a**) and magnetic (**b**) impurity in a chiral *d*-wave superconductor at finite doping away from half-filling (|*μ*| = 0.1). Here Δ_*d*_/Δ_*d*′_ = 2 and with the same vertical and horizontal axes as in Fig. 2.

If the particle-hole symmetry of the normal states is broken, here by setting 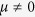, then the summation over the bare Green’s function also contains *F*_3_*τ*_3_*σ*_0_, where, up to first order in 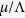, 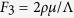. The influence of this new term on the bound states is much more pronounced for the chiral *d*-wave state compared to the *d* + *is*-wave state. For a potential impurity the bound states energies are still two-fold degenerate, but a non-zero *μ* shifts the energy of the bound states in the unitary limit, as is illustrated in Fig. 3(a). In fact, for the electron doped case, *μ* > 0, a level crossing now appears for repulsive impurity scattering (*U*_*pot*_ > 0), while for a hole doped system, *μ* < 0, the level crossing instead occurs for an attractive impurity (*U*_*pot*_ < 0). Thus doping can be used as a simple means to control the level crossing for potential impurities. A local chemical potential induced by a tunneling probe could even offer *in*-*situ* tunability of the level crossing^11^.

For a magnetic impurity the bound state degeneracy is lifted for finite *μ*, as illustrated in Fig. 3(b) for positive *U*_*mag*_. In this case, one pair of bound states move away from the middle of the gap (thick red lines). For these states no zero modes are thus expected even in the unitary scattering limit for any positive scattering *U*_*mag*_ > 0. The other pair of bound states (thin blue lines) move toward the middle of the gap and consequently a level crossing appears at some 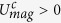. The bound state energy spectrum is symmetric with respect to 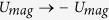, and thus there is another level crossing at the corresponding negative magnetic scattering. In addition to being symmetric with respect to the sign of *U*_*mag*_, the bound states also do not depend on the sign of *μ*. Remarkably, the dimensionless critical coupling for reaching a zero-energy state for both magnetic and potential scatterers is the same 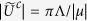. As seen, this critical coupling can be decreased by increasing |*μ*|.

For the purpose of the forthcoming self-consistent numerical calculation, we mention already here that even at half-filling, the energy degeneracy can still be lifted by adding a small amount of extended *s*-wave superconductivity to the chiral *d*-wave. More precisely, if the order parameter takes the form 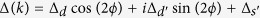, where Δ_*s*′_ is *k*-independent, the energy degeneracy for magnetic impurity bound states is lifted, while the bound states remain degenerate for a potential impurity. Therefore, the degeneracy of the bound states in the presence of a magnetic impurity in a chiral *d*-wave superconductor is very fragile and can easily be lifted.

### Numeric tight-binding lattice calculation

We now turn to discuss the results obtained from tight-binding lattice calculations. In all calculations we use a generic finite-size square lattice in which we consider a single impurity located at the middle site. Again we consider both a *d* + *is*- and chiral *d* + *id*′-wave superconductor. The effective Hamiltonian for the 2D superconducting host with an impurity with both potential and magnetic scattering elements located at *R* is refs 23,31


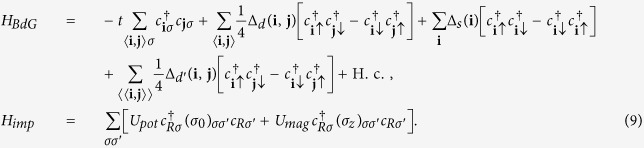


Here **i** = (*i*_*x*_, *i*_*y*_) represents a site in the square lattice, with the lattice spacing *a* set to be 1. The dominant *d*-wave order exists on nearest neighbour bonds, while the subdominant *s*-wave order is an on-site parameter and the *d*′-wave order reigns on next nearest neighbour bonds. For the self-consistent calculations (see below) we do not a priori assume any symmetries or conditions for any of these three order parameters. However, in calculations with constant order parameters throughout the sample, i.e. non-self-consistent calculations, we enforce the *d*-wave order by setting 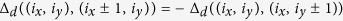 for all sites, i.e. the order parameter on *y*-directed bonds is equal in magnitude but with opposite sign compared to the order parameter on *x*-directed bonds. Likewise, the *d*′-wave state has opposite signs on bonds in the ±(*x* + *y*) direction compared to bonds in the ±(*x* − *y*) direction. We also enforce the subdominant order parameter (*s* or *d*′) to be purely imaginary in the non-self-consistent calculations.

We solve Eq. (9) by performing a Chebyshev polynomial expansion of the corresponding Green’s function^32^,^33^,^34^. This method allows us to investigate lattices with very large number of lattice points because the amount of necessary computational resources grow only linearly with the size of the system, far outperforming regular diagonalization. More specifically, we calculate the Green’s function for the impurity site and its closest neighbours. The imaginary part of Green’s function gives the local density of states (LDOS) and the bound states are easily identified as sharp peaks inside the energy gap at the impurity site and also its neighbouring sites.

**Figure 4:**
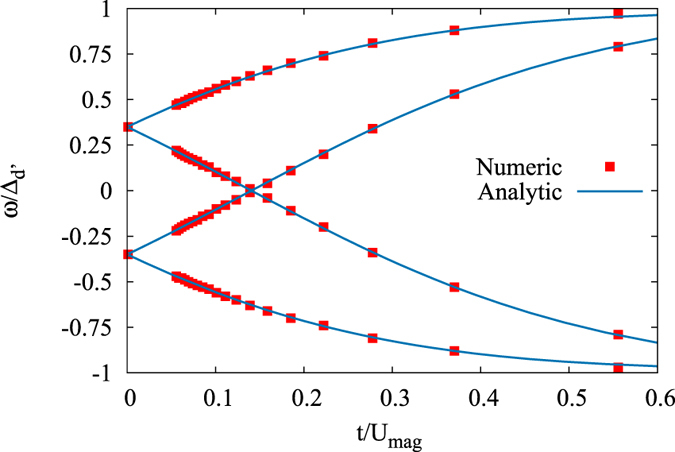
The energy spectrum of all intra-gap bound states for a magnetic impurity in a chiral *d*-wave superconductor as function of inverse of impurity strength 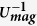. The order parameters are set to Δ_*d*′_/*t* = 0.05, Δ_*d*_/Δ_*d*′_ = 2, and *μ/t* = 0.5, and with lattice size 201 × 201.

We find that the tight-binding calculations fit exactly to the analytical T-matrix results. For instance, in Fig. 4 we show the bound state spectrum generated by a magnetic impurity in a chiral *d*-wave superconductor at finite doping, for both the numeric tight-binding method and an analytic T-matrix calculation. In order to be able to do this comparison, we evaluate the summations appearing in the T-matrix formalism for a discrete mesh over the first Brillouin zone of the square lattice using the same form of the kinetic energy. The main difference between the continuum and discrete calculation is that in the former we neglect the radial dependence of the order parameter in reciprocal space while in the latter, we keep both the radial and angular dependence. Therefore, the discrete summation over the full tight-binding model gives us a more relevant solution to compare with the finite-size numerical calculations. We also find an excellent agreement in the unitary scattering limit (*U*_*mag*_ → ∞), which in the tight-binding lattice calculation can be implemented by simply removing the impurity site, thus creating a vacancy.

### Self-consistent results

Above we simply assumed constant order parameters and enforced the correct condensate symmetries. Now we allow the condensate to appropriately respond to the impurity through a proper self-consistent calculation. Since the superconductor symmetry is important for the properties of the bound states, this is the most accurate way to ensure a correct solution. In these self-consistent calculations we only assume a finite and constant pair potential *V* in each pairing channel and calculate the order parameter(s) explicitly everywhere in the lattice. For a *d*-wave state we use the self-consistent condition 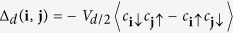, where **i**, **j** are nearest neighbour sites. In the self-consistent calculation we start by guessing a value for Δ_*d*_ on each bond, solve Eq. (9), calculate a new Δ_*d*_ on each bond using the self-consistent condition, and repeat until Δ_*d*_ does not change between two subsequent iterations. For the *d* + *is*-wave state we also assume a finite *V*_*s*_ in addition to *V*_*d*_ and calculate separately 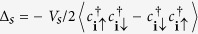 self-consistently. For the *d* + *id*′-wave state 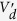 is likewise finite, such that 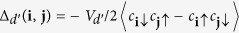, where **i**, **j** are next nearest neighbour sites.

We take the initial guess for the subdominant order parameter to be purely imaginary but through the self-consistency loop it is free to acquire a real component as well. Likewise, we emphasize that Δ_*d*_ on *x*- and *y*-directed bonds are treated fully independent and the same applies to the *d*′-wave state. Thus, we have not a priori assumed any symmetry for any of the pairing states. In the calculations we use *V*_*d*_/*t* = 1.7, *V*_*s*_/*t* = 1.7 for *d* + *is*-wave state and *V*_*d*_/*t* = 1.8, *V*_*d*′_/*t* = 1.7 for chiral *d*-wave state, but the results are not sensitive to these particular values. We also set *μ/t* = −1 for the most general case and to avoid the van Hove singularity at half-filling. We mainly use a 51 × 51 lattice, with similar results obtained with a 31 × 31 lattice, which guarantees that the results are not sensitive to the lattice size.

**Figure 5:**
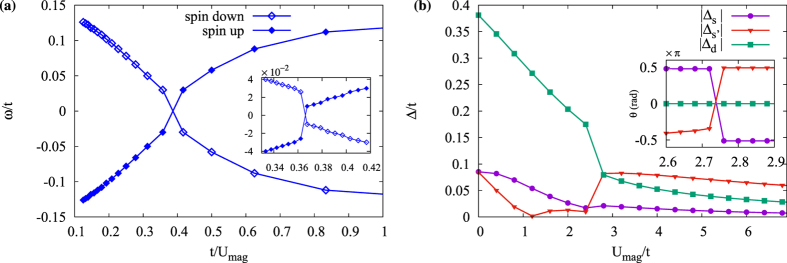
The energy spectrum of all intra-gap bound states (**a**) and the order parameters found self-consistently at the impurity site (**b**) for a magnetic impurity in a 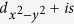-wave superconductor as a function of impurity strength. Insets shows zoom-ins around the critical point where the zero-energy level crossing, with the inset in (**b**) showing the phase of the order parameters only.

Using the self-consistently calculated order parameters, we extract the LDOS at and close to the impurity site for both *d* + *is*- and *d* + *id*′-wave states in the presence of either magnetic or potential impurities. The self-consistent tight-binding lattice calculations reveal that the results obtained for fixed order parameters are still largely valid. However, important effects appear around the critical scattering strength in the self-consistent calculations. Starting with the *d* + *is*-wave state, the self-consistent results show that the intra-gap localized bound states from a magnetic impurity behave largely in a similar way to their non-self-consistent counterpart as seen in Fig. 5(a). The main discrepancy is close to the critical scattering 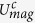. As seen in the inset in Fig. 5(a), the energy of the bound states does not evolve smoothly at the transition point and there is instead a clear kink at 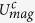. This is a finger print of a first-order quantum phase transition. A similar effect has been found in a pure *s*-wave superconductor^30^.

The order parameter at the impurity site shed more light on this transition as can be seen in Fig. 5(b), where sudden changes near the critical coupling are visible. Self-consistently calculating the order parameters, there is in addition to the on-site *s*-wave and 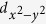-wave order parameters, also an extended *s*-wave order parameter. This extended *s*-wave order resides on the nearest neighbour bonds and appears only very close to the impurity. It is thus a direct consequence of the impurity weakening the *d*-wave character in favour of the more disorder-robust *s*-wave symmetry. At the quantum critical point this extended *s*-wave state even becomes the dominant order parameter but notably only at the impurity site, farther away the *d*-wave order parameter is still dominant. In addition, both the *s*-wave and extended *s*-wave order parameters develop a *π*-phase shift on the impurity site across the critical coupling. This is in line with previous calculations for pure *s*-wave superconductors where the *s*-wave state undergoes a local *π*-shift^15^,^30^,^35^. However, note that we find that the phase of the dominant *d*-wave state stays constant.

**Figure 6:**
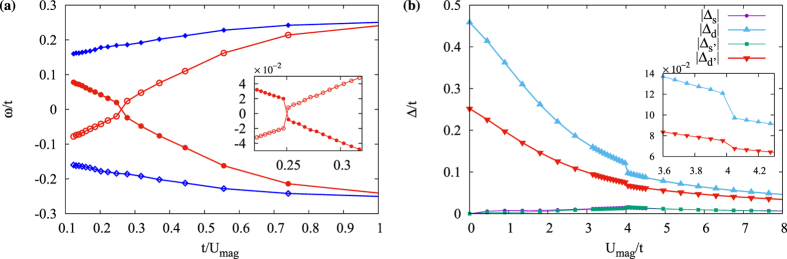
The energy spectrum of all intra-gap bound states (**a**) and the order parameters found self-consistently at the impurity site (**b**) for a magnetic impurity in a 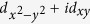-wave superconductor states as function of impurity strength. Insets shows zoom-ins around the critical point where the energy levels cross zero.

For the case of a magnetic impurity in a chiral *d*-wave superconductor, there is only a level crossing for one pair of bound states as seen in Fig. 6(a), as also found in the non-self-consistent tight-binding and T-matrix calculations. The self-consistent solution, however, shows a kink close to the critical scattering, signaling a first-order phase transition as in the *d* + *is* case. Considering the order parameter, the self-consistent calculation reveals that the dominant 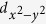 stays the dominant order parameter even beyond the critical scattering and the subdominant state is also always the *d*_*xy*_-wave state, as seen in in Fig. 6(b). In this case only very weak extended *s*-wave components appear on nearest and next-nearest neighbour bonds, defined here as Δ_*s*_ and Δ_*s*′_, respectively. Interestingly, this means that for a chiral *d*-wave superconductor, the impurity does not disturb the dominant *d*-wave orders nearly as much as in the *d* + *is*-wave case. Despite the smallness of the *s*-wave components generated in the self-consistent calculations, we find that they are still responsible for lifting of the degeneracy of the impurity bands in the half-filled lattice case (*μ* = 0).

## Conclusions

In this work we have investigated impurity-induced bound states in fully gapped *d*-wave superconductors. The main results are summarized in Table 1. As illustrated by this table, we have shown that an impurity, whether magnetic or potential, induces two pairs of intra-gap bound states in a chiral *d* + *id*′-wave superconductor, while for a *d* + *is*-wave superconductor, there is only one (zero) pair of bound states for a magnetic (potential) impurity. As a result, the number of intra-gap bound states becomes a powerful means for establishing the symmetry of the superconducting state in a fully gapped *d*-wave superconductor, such as that recently established in cuprate nanoislands or at certain cuprate surfaces^22^,^25^. With potential impurities also tunable by localized potential scattering from a tunneling probe^11^, there even exist possibilities to study the evolution of the bound states of a particular impurity for a range of effective impurity strengths.

Another important difference between *d* + *is*-wave and chiral *d*-wave superconductors is the behaviour of the zero-energy level crossings for the impurity-induced states. For a *d* + *is*-wave superconductor, increasing the magnetic scattering strength *U*_*mag*_ leads to a level crossing of the bound states, which means there always exists a critical coupling 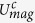, separating two distinct ground states. However, for a chiral *d* + *id*′-wave superconductor there is no level crossing for a particle-hole symmetric normal band structure (here indicated by *μ* = 0) at any finite scattering strength, for either potential or magnetic impurities. Only at significant doping away from *μ* = 0 there is a zero-energy level crossing at an experimentally achievable scattering strength. It is also important to notice that the impurity bound states in a chiral *d* + *id*′-wave superconductor are often twofold degenerate for both potential and magnetic impurities. For a magnetic impurity the degeneracy is lifted by either a finite *μ* or by ever-present subdominant extended *s*-wave components, as we find in our self-consistent calculations, nonetheless, there are often two nearly degenerate states.

The systems with zero energy states and no impurity band degeneracies, i.e. magnetic impurities in both *d* + *is*- and *d* + *id*′-wave superconductors, have the potential for hosting Majorana fermions. However, in the case of a single impurity, the two zero energy states at 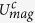 occupy the same point in space (the impurity site) and thus these Majorana fermions will simply combine to form a regular electron quasiparticle excitation. This is why 1D magnetic impurity wires or other higher dimensional objects are needed to spatially separate the Majorana fermions and harness their Majorana character. Moreover, at least a small spin-orbit coupling has been shown to be essential in both *s*-wave^4^,^5^,^6^,^36^ and *d* + *id*′-wave^31^,^37^ superconductors in order to generate a non-trivial topological phase. In the chiral *d* + *id*′-wave superconductor the near degeneracy of the impurity bands however possess additional complications for Majorana fermions in an impurity wire. An even number of positive (or negative) near zero-energy states in the single impurity limit will result in two putative Majorana end modes for a wire, which then hybridize and split off from zero energy, losing their Majorana character. Thus, in terms of the potential for generating Majorana modes, our results directly shows that only magnetic impurity wires in a *d* + *is*-wave superconductor or in a heavily doped *d* + *id*′-wave state are promising systems.

Finally, we emphasize that our self-consistent calculations confirm our analytical results where we have assumed constant order parameters uninfluenced by the impurities. In addition, the self-consistent calculations shed more light on the nature of the zero-energy level crossings and show that for both *d* + *is* and *d* + *id*′-wave superconductors, these are first-order quantum phase transitions, with clear discontinuities in both energy levels and order parameters. For the *d* + *is*-wave state we even find a local *π*-shift at the phase transition for all subdominant order parameters, consistent with the behaviour in conventional *s*-wave superconductors^30^,^35^. For the *d* + *id*′-wave superconductor we, however, do not find any *π*-shifts.

## Additional Information

**How to cite this article:** Mashkoori, M. *et al*. Impurity bound states in fully gapped *d*-wave superconductors with subdominant order parameters. *Sci. Rep.* **7**, 44107; doi: 10.1038/srep44107 (2017).

**Publisher's note:** Springer Nature remains neutral with regard to jurisdictional claims in published maps and institutional affiliations.

## Figures and Tables

**Table 1:** 

	*d* + *is*	*d* + *id*′ (*μ* = 0)	*d* + *id*′ (*μ* ≠ 0)
*U*_*pot*_	×	2 × 2	2 × 2
*U*_*mag*_	2	2 × 2	4
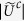	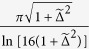	∞	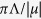
*π*-shift	✓	×	×

The number of bound states and critical scattering strengths for impurities in *d* + *is*-wave and *d* + *id*′-wave superconductors. 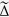 represents the ratio of dominant over subdominant order parameter, while 2 × 2 indicates two states being two-fold degenerate.
